# The *plk1* Gene Regulatory Network Modeling Identifies Three Circuits for *plk1*-mediated Genomic Instability Leading to Neoplastic Transformation

**DOI:** 10.3390/life15050799

**Published:** 2025-05-17

**Authors:** Jeison F. Suescum-Holguín, Diana Carolina Clavijo-Buriticá, Edward Fabian Carrillo-Borda, Mauricio Alberto Quimbaya

**Affiliations:** 1 Facultad de Ingeniería y Ciencias, Instituto de Investigaciones en Ciencias Ómicas iÓMICAS, Pontificia Universidad Javeriana Cali, Santiago de Cali 760031, Colombia; jason5h@javerianacali.edu.co (J.F.S.-H.); diana.clavijo@javerianacali.edu.co (D.C.C.-B.); 2 Grupo de Investigación en Microbiología Molecular y Enfermedades Infecciosas (GIMMEIN), Faculty of Health Sciences, Universidad Libre Seccional Cali, Santiago de Cali 760043, Colombia; edwardf-carrillob@unilibre.edu.co

**Keywords:** polo-like kinase regulatory network, cancer systems biology, genomic instability, genome instability mathematical model and novel cancer markers

## Abstract

Genomic instability has been increasingly recognized over the past decade as a fundamental driver of cancer initiation and progression, largely owing to its association with specific genes and cellular mechanisms that offer therapeutic potential. However, a comprehensive molecular framework that captures the interconnected processes underlying this phenomenon remains elusive. In this study, we focused on polo-like kinase 1 (PLK1), a key cell cycle regulator frequently overexpressed in diverse human tumors, to reconstruct a regulatory network that consolidates pre-existing biological knowledge exclusively related to pathways involved in genome stability maintenance and cancer. The resulting model integrates nine biological processes, 1030 reactions, and 716 molecular species to form a literature-supported network in which PLK1 serves as a central regulatory node. However, rather than depicting an isolated PLK1-centric system, this network reflects a broader and more complex architecture of interrelated genomic instability mechanisms. As expected, the simulations reproduced known behaviors associated with PLK1 dysregulation, reinforcing the well-established role of the kinase in genome destabilization. Importantly, this model also enables the exploration of additional, less-characterized dynamics, including the potential involvement of genes such as *kif2c*, *incenp*, and other regulators of chromosomal segregation and DNA repair, which appear to contribute to instability events downstream of PLK1. While these findings are grounded in mechanistic simulations and require further experimental validation, gene expression and survival analyses across tumor types support their clinical relevance by linking them to poor prognosis in specific cancers. Overall, the model provides a systemic and adaptable foundation for studying PLK1-related genomic instability, enabling both the reinforcement of known mechanisms and discovery of candidate genes and circuits that may drive tumorigenesis through compromised genome integrity across distinct cancer contexts.

## 1. Introduction

Genomic instability is a defining hallmark of cancer, underpinning the progressive accumulation of mutations, chromosomal rearrangements, and aneuploidy that drives tumorigenesis and therapy resistance [1]. This instability results from the breakdown of the cellular mechanisms responsible for preserving genome integrity, including DNA repair, mitotic checkpoint control, and chromosomal segregation [2,3,4,5]. Among the molecular regulators involved, Polo-like kinase 1 (PLK1) has been identified as a central orchestrator of mitotic progression and genome maintenance. PLK1 regulates processes such as centrosome maturation, bipolar spindle formation, kinetochore-microtubule attachment, and cytokinesis, and its dysregulation has been consistently linked to mitotic errors and chromosomal missegregation in a variety of cancer types [6].

Despite a growing body of experimental evidence on the functions of PLK1, our understanding of its role as a driver of genomic instability remains fragmented and largely descriptive. Current knowledge tends to be scattered across individual studies focusing on isolated molecular interactions or specific biological contexts, limiting our ability to infer its systemic behavior under perturbed conditions such as overexpression. This limitation is particularly relevant given that PLK1 is frequently upregulated in tumors, and its overexpression has been associated with poor clinical outcomes [7].

In recent years, computational systems biology has emerged as a powerful approach for integrating dispersed biological data and reconstructing complex molecular systems in silico. This paradigm enables researchers to model tumorigenic processes holistically by creating mathematical representations that combine theoretical and experimental knowledge into cohesive, analyzable frameworks [8]. Numerous models have been developed to simulate essential cellular processes including cell cycle regulation, apoptosis, DNA damage response, and other disease-related pathways [9,10,11,12]. Specific efforts have been made to model components directly related to genome stability, such as DNA surveillance mechanisms and cell cycle checkpoints [13,14], or to describe genomic instability using abstract event-based models [15]. Although PLK1 has been incorporated into several of these models [16,17], its role as a potential initiator or amplifier of genomic instability has not yet been systematically explored within a dedicated regulatory framework.

In this study, we addressed this gap by proposing a comprehensive system-level reconstruction of the PLK1 regulatory network in the context of genomic instability. We systematically compiled and curated information from literature and databases to model the upstream regulators, downstream targets, and functional partners of PLK1, focusing on their collective influence on genomic integrity. This reconstruction was implemented into a dynamic computational model that captured the behavior of the system under different regulatory conditions, including PLK1 overexpression.

The resulting model not only recapitulates behaviors consistent with experimental observations, but also enables the prediction of novel interactions and regulatory consequences that may underlie PLK1-mediated chromosomal instability. In doing so, the model serves as a framework for hypothesis generation, offering insights into the understudied genes and proteins that respond to perturbations in PLK1 signaling. Although computational predictions are based on experimentally derived regulatory information, they highlight potential targets for future validation in the context of cancer biology.

By bridging fragmented knowledge through system modeling, this study contributes to a deeper understanding of the molecular underpinnings of genomic instability and positions PLK1 as a central node in this complex landscape.

## 2. Materials and Methods

### 2.1. Retrieving Genomic Instability-Associated Biological Processes Using PathwayStudio

PathwayStudio (v12.3) is supported by a comprehensive large mammal-centered database that includes 93,000 items, such as genes, proteins, biomarkers, and more than 11 million relationships between these objects. The tool provides access to the database through a search tool called MedScan, which utilizes natural language processing technology to retrieve and interpret information from the database within a specific context [18] https://mammal-profservices.pathwaystudio.com/app/search (accessed on 16 January 2024).

A search was performed in PathwayStudio using the PLK1 protein identifier and specific search filters to search for curated pathways related to diseases and cellular processes. The cellular processes most relevant to genomic instability and cancer were selected based on available literature [1,19,20,21,22,23,24]. The selected biological processes were composed of various biological interactions, including [i] protein–protein interactions, [ii] protein-protein complex interactions, [iii] protein-small-molecule interactions, and [iv] protein-end biological process interactions. The latter interaction type describes the relationship between proteins and the progress of other biological processes that are not necessarily related to genomic instability. Each interaction was manually curated to validate the interpretation provided by PathwayStudio and to fill any information gaps left by the tool (More details in Section 4.1). The curation process involved using the search sentences provided by PathwayStudio, associated articles, GeneCards database [25], and supplementary literature.

### 2.2. Delimiting the Core of plk1- Associated Genomic Instability Events

**Figure 1:**
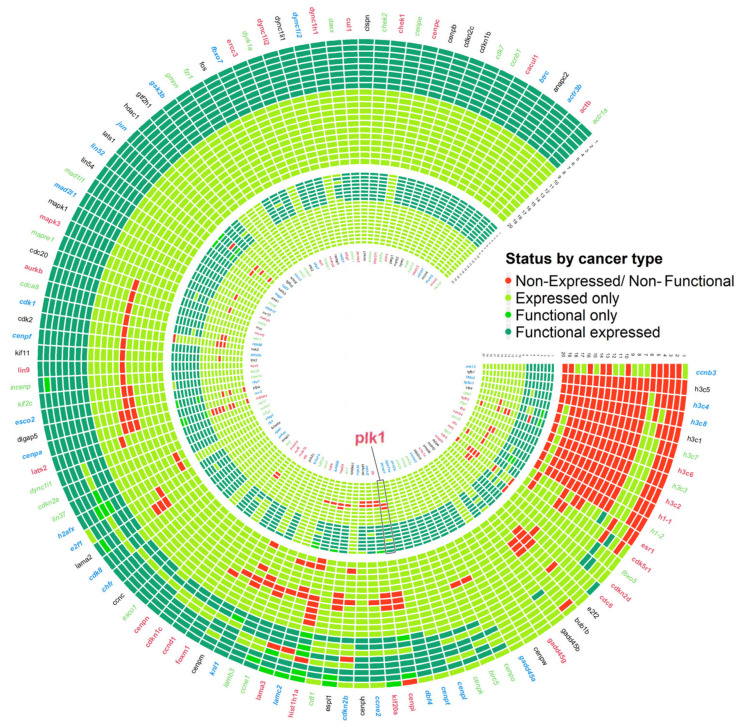
An expression matrix for 220 protein-encoding genes was linked to nine biological processes related to PLK1-mediated genomic instability across various cancer types. The cancer types are numbered from the outermost circle to the innermost one as follows: (1) Hepatocellular Carcinoma, (2) Lung Squamous Cell Carcinoma, (3) Lung Adenocarcinoma, (4) Glioblastoma Multiforme, (5) Anaplastic Astrocytoma, (6) Colorectal Carcinoma, (7) Uterine Corpus Endometrial Carcinoma, (8) Pancreatic Ductal Adenocarcinoma, (9) Triple-Negative Breast Cancer, (10) Non-Small Cell Lung Cancer, (11) Stomach Adenocarcinoma, (12) Merkel Cell Carcinoma, (13) Basal Cell Carcinoma, (14) Mixed Phenotype Acute Leukemia, (15) Neuroblastoma, (16) Muscle-Invasive Urothelial Bladder Cancer, (17) Ovarian Carcinoma, (18) Anaplastic Thyroid Carcinoma, (19) Acute Myeloid Leukemia, (20) Ductal Carcinoma In Situ. The inner circle follows the same pattern.

We isolated the gene expression list of 20 cancer single-cell transcriptomic datasets available in CancerSCEM [24] to build a database of genes expressed in each type of cancer. Additionally, proteomic datasets for eight out of the 20 cancers were retrieved from the Proteomic Data Commons (PDC) (https://pdc.cancer.gov/, accessed on 7 June 2024). and utilized to validate functional gene expression. Each gene in the previous step in the biological processes identified using PathwayStudio was contrasted against the database derived from cancer transcriptomic assays to determine whether it was functionally expressed across a wide spectrum of tumors. We defined a threshold of inclusion (the gene was expressed at least of 75% tumor datasets) to filter and conserve only those active genes in most of the considered cancer types, resulting in a condensed list of expressing/functionally expressing genes related to genomic instability events. By clearly documenting this cutoff (75% of tumors), we make the gene inclusion process transparent and reproducible. Notably, this threshold captures approximately 92% of the 220 unique genes identified (Figure 1), indicating that the vast majority of relevant genes are retained, thereby balancing inclusiveness with stringency

The cancer types considered for the database were: HCC *: Hepatocellular Carcinoma, LUSC *: Lung Squamous Cell Carcinoma, LUAD *: Lung Adenocarcinoma, TNBC: Triple-Negative Breast Cancer, NSCLC: Non-Small Cell Lung Cancer, STAD: Stomach Adenocarcinoma, GBM *: Glioblastoma Multiforme, AA: Anaplastic Astrocytoma, MCC: Merkel Cell Carcinoma, CRC *: Colorectal Carcinoma, BCC: Basal Cell Carcinoma, MPAL: Mixed Phenotype Acute Leukemia, NBL: Neuroblastoma, MIUBC: Muscle-Invasive Urothelial Bladder Cancer, UCEC *: Uterine Corpus Endometrial Carcinoma, OV: Ovarian Carcinoma, ATC: Anaplastic Thyroid Carcinoma, AML *: Acute Myeloid Leukemia, PDAC *: Pancreatic Ductal Adenocarcinoma, DCIS: Ductal Carcinoma In Situ. The cancer types marked with an asterisk (*) also have an associated proteomic profile.

### 2.3. Construction of the plk1 Core Genomic Instability Network

The processes, biological species, and their respective interactions identified and filtered in the previous steps were then employed to construct the model representing the *plk1* Core Genomic Instability Network. This was performed using CellDesigner software (v4.4.2), a widely used tool in systems biology for the construction and simulation of biological models. The model was created in the standard SBML [Systems Biology Markup Language] format [26]. Additionally, a dictionary compilation was necessary to translate the graphical notation from PathwayStudio to CellDesigner following the SBGN [Systems Biology Graphical Notation] criteria proposed in the software user manuals and the consensus generated for this work.

The model was built considering the following considerations: [i] for each protein species in the original network provided by PathwayStudio, the corresponding gene and messenger RNA [mRNA] were added to model the entire process of genetic information flow [transcription and translation]; [ii] to represent the enzymatic activity, four modification types were included: phosphorylation, dephosphorylation, ubiquitination, and acetylation; [iii] all protein components of the network were assigned a degradation reaction to complete the mass balance in the mathematical model. The CellDesigner software has different reaction types, such as transcription, translation, and state transition reactions, which are assigned according to each case. Finally, the unification of all processes into a single network is possible because of the shared reactions among biological processes. Redundant reactions and biological species were eliminated. Duplication of molecules and proteins between processes was avoided by assuming a single global version participating in the entire network.

### 2.4. Mathematical Modeling and Generation of Relevant Simulation Scenarios

A deterministic approach was adopted with elementary kinetics under the law of mass action to generate a system of differential equations [ODEs] to model the constructed network in CellDesigner [27]. The model made the following assumptions: [i] depreciation of the cell dilution factor; [ii] sufficient and available concentration of genes, molecules, and proteins at the cellular level for species in which the synthesis process in the model is not considered; [iii] transport reactions are not included, and global reactions occurring are assumed, regardless of spatial separation.

The kinetic parameters required for each reaction in the regulatory network were meticulously searched in specialized biological databases, such as Biomodels [28], BRENDA [29], and Sabio-RK [30], and were complemented with extensive searches in the scientific literature. Reactions for which kinetic constant values were not reported in the literature were assigned based on the published kinetic values for reactions of the same type (more details in Section 4.1). Finally, the units of measurement for all parameters were unified in micromolar [µM] and the time units in hours [h^−1^]. The reactions (Table S2) and parameter lists (Table S3) are available at: https://data.mendeley.com/datasets/xjwm5ygymn (accessed on 13 May 2025).

Simulations of the *plk1* Core Genomic Instability Model were performed using the SBML ODE solver library [SOSlib] in CellDesigner v.4.4.2 [26]. Simulations were performed for 150 intervals, corresponding to 0.28 h. An equivalence of 85-time intervals defined the duration of each interval for the modeled biological system to reach a steady state and the course of the mammalian cell cycle, as explained in the results. The 100 integration steps and default CellDesigner solver, SOSlib, were maintained. The initial concentration [IC] for all genes was 1.0 µM to recreate homogeneous conditions for basal expression of all genes, initialize the model, and assess the overall dynamics of the network. In addition, the proteins we considered present in the free form in the model had an initial concentration of 5.0 µM. These uniform starting values are standard assumptions to reflect baseline gene expression and protein availability in the cell. Subsequently, different simulation scenarios were considered to evaluate the response of the model to different types of perturbations. We chose to change the concentration of the biological species with the highest number of interactions in the network (CDK1, COHESIN, and PLK1) (Table 1) because these species are known to play crucial roles in network dynamics, and their perturbation could have significant effects on the system.

### 2.5. Transcriptomic Profiling and Survival Analysis

Transcriptomic profiles and survival plots were built using the GEPIA2 database [31]. The differences in the expression levels of the genes comprising the circuits were calculated using Log2FoldChange of The Cancer Genome Atlas (TCGA) for each cancer type vs. Genotype-Tissue Expression (GTEx).

### 2.6. Cell Culture and Quantitative Real-Time Polymerase Chain Reaction

HEK293 (embryonic kidney) and HT-29 (colon adenocarcinoma) cell lines were used. Both cell lines were cultured in DMEM supplemented with 10% fetal bovine serum, 1% L-glutamine, and 1% penicillin-streptomycin. For both cell lines, approximately 100,000 cells were seeded in 4 mL of medium in a 25 cm^2^ culture flask and incubated at 37 °C with 5% CO_2_ until the cultures reached 80% confluence. At that time, the necessary passages were performed to ensure the survival and viability of the culture for the duration of the experiment. Both cell lines were purchased from ATCC; HEK293 catalog CRL-1573 and HT29 catalog HTB-38.

The RNeasy Mini Kit (Qiagen, Germantown, MD, USA) was used for RNA extraction according to the manufacturer’s instructions. The quantity and quality of the extracted RNA were determined using a NanoPhotometer N120 (Implen GmbH, Munich, Germany). Subsequently, 1 μg of RNA was converted into complementary DNA (cDNA) using the Transcriptor Universal cDNA Master Mix kit (Merck S.A., Darmstadt, Germany) according to the manufacturer’s instructions. Based on the proposed circuits and mechanisms by which PLK1 can regulate genomic instability events, we selected targets to validate the influence of PLK1 on the proposed mechanism and genes. Thus, qPCR targeted *plk1*, *aurkb, incenp, kif2c* and *pttg1*. Real-time qPCR analyses were carried out in QuantStudio 6 Pro (Thermo Fisher™, USA). As a template, 6 ng of each cDNA sample was amplified in 10 µL of reaction mix buffer containing 5 µL of SYBR^®^ Green qPCR Ready mix™ (Merck S.A., Darmstadt, Germany) and forward and reverse primers at a final concentration of 2.5 µM. The reaction mixtures contained the intercalating dye SYBR Green I for cDNA detection and analysis. The thermal cycling protocol involved a pre-incubation step at 95 °C for 10 min followed by three amplification steps of 45 cycles (95 °C for 30 s, 62 °C for 30 s and 72 °C for 35 s). The program ended with Tm calling analysis necessary for calculating the melting curves and peaks. The matpsy and actin genes were used as normalization factors for all experiments. The primer sequences followed in this study are available at (https://data.mendeley.com/datasets/xjwm5ygymn (accessed on 13 May 2025)).

Three independent experiments were carried out to analyze relative gene expression, and each sample was tested in quadruplicate. The expression levels were analyzed using qbase+ 3.4 (Biogazelle, Zwijnaarde, Ghent, Belgium; www.qbaseplus.com (accessed on 13 May 2025)). The real-time value for each sample was averaged and compared using the Ct method and normalized to endogenous controls, matpsy, and actin. The relative expression levels were calibrated at 1.0, using HEK-293 as a reference. We used an unpaired t-test in the qbase+ statistical package to calculate the mean differences.

## 3. Results

### 3.1. Identifying and Integrating the Key Components of plk1-Mediated Genomic Instability Events in Cancer

The biological processes retrieved from the PathwayStudio database were reviewed to determine their relationship with genomic instability events, based on previous associations reported by different authors [1,19,20,22,23,24]. In addition, we confirmed that the reported evidence on the mechanisms, genes and particular pathways of genomic instability related to PLK1 was included in the retrieved data and completely covered by the selected biological processes [6]. Overall, nine biological processes related to genomic instability were identified, in which PLK1 plays a role (Table 2). The processes were grouped into three categories assigned by PathwayStudio search: [i] cellular processes describing mitotic events and cell cycle checkpoints closely linked to the machinery that enables proper chromosome segregation and maintenance of chromosomal stability; [ii] disease-associated processes and [iii] a pathological process describing signaling pathways in cancer involving FOXM1, a transcription factor that regulates proliferation and cell cycle checkpoints [32]. PLK1 serves as a pivotal protein in all selected biological processes. Its critical role in regulating the cell cycle makes it a key driver of essential pathways that remain active in both healthy and carcinogenic contexts. This centrality to cell cycle control underscores why the processes chosen around PLK1 are indispensable for maintaining cellular function, regardless of pathological conditions.

The nine biological processes comprised 568 properly curated interactions between 378 biological species, mostly consisting of proteins and protein complexes. For each protein within the 378 biological species, the respective coding gene symbols (HGNC and synonyms) were identified. Similarly, in the case of protein complexes, subunit-coding genes were retrieved. In total, 220 unique genes were obtained after removing repeated species among the biological processes.

To determine the core molecular machinery associated with *plk1*-mediated genome instability events, unique genes were compared against the CancerSCEM database [33], a repository containing gene expression profiles generated from single-cell transcriptomic assays derived from 20 different cancer types. Additionally, these profiles were complemented with the proteomic signature of the analyzed carcinomas obtained from the Proteomic Data Commons (PDC) database. Genes reported to be not expressed in most cancer types primarily correspond to histone-coding genes. These genes lack a polyA sequence in their mRNA, rendering them undetectable using RNA-seq techniques [34].

More than 82% of the evaluated genes were expressed in at least 19 cancer types; additionally, 8 cancer types with an available proteomic profile showed similar results, with an average of 77% of the genes reporting the functional expression of their associated proteins. We considered as the core machinery associated with the genome instability hallmark genes that were expressed in at least 16 out of the 20 evaluated cancer types, which accounted for approximately 92% of the detected unique genes (Figure 1).

**Figure A1:**
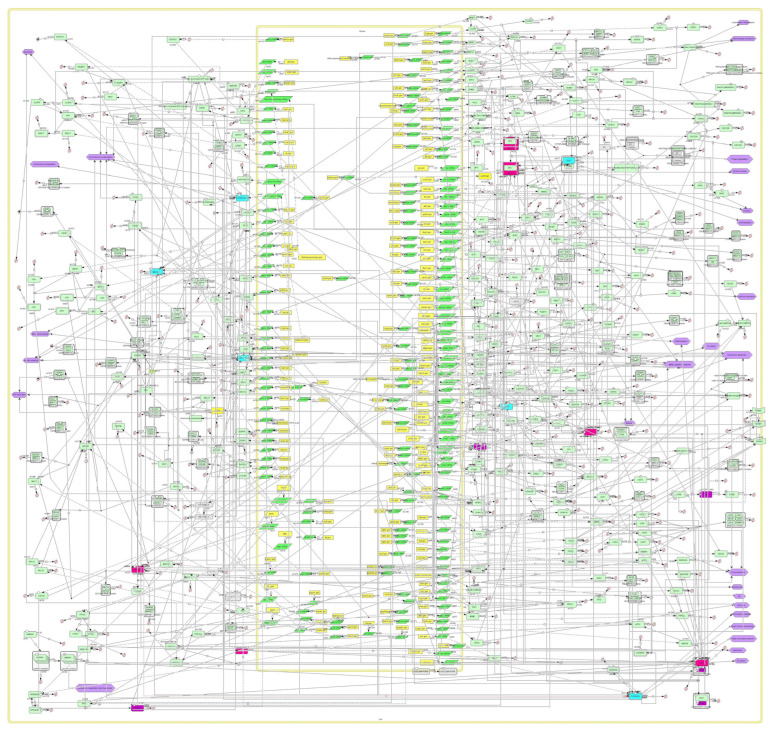
Graphical summary of the *plk1* gene regulatory network. Cell and nuclear compartments are shown. All genes and mRNA inside the nucleus are represented as yellow rectangles and green rhomboids, respectively, the proteins are represented as rounded rectangles. Gray rounded rectangles containing proteins inside represent protein complexes; furthermore, pink and cyan colored proteins differentiate highly interactive proteins. Finally, purple shapes represent external biological processes or particular cell events regulated by proteins present in the network.

Analysis of the interactome derived from retained proteins/genes revealed that CDK1, as part of the MPF complex, displayed the highest number of interactions, with a total of 43 connections. Similarly, the COHESIN complex (pds5a, rad21, smc1a, smc3) and PLK1 were hubs in the generated network, with 23 interactions each (Appendix A, Figure A1). Genes encoding the CDK1, PLK1, and COHESIN subunits RAD21 and SMC1A have been previously linked to genomic instability events [3,35,36]. Additionally, the network included 59 other genes associated with genomic instability or chromosomal aberrations, accounting for approximately 32% of the total genes retained. The final gene list, along with the interactions retrieved from PathwayStudio, was integrated to generate the Plk1 core genomic instability network.

### 3.2. Building the plk1 Core Genomic Instability Network

We used CellDesigner software as the network building platform and adapted the graphical notation of PathwayStudio (RNEF) into CellDesigner Systems Biology Standard Graphical Notation (SBGN). The shared species and interactions among the biological processes served as anchor points to combine the nine processes. Additionally, we decided to extend the network and include the complete genetic information flux from genes to proteins. The generated network consisted of 716 biological species of different types, including 185 genes, 185 mRNAs, 247 proteins, 70 protein complexes, and 28 end-biological processes (Table S1, https://data.mendeley.com/datasets/xjwm5ygymn (accessed on 13 May 2025)), generating 1030 reactions of different types (Table S2, https://data.mendeley.com/datasets/xjwm5ygymn (accessed on 13 May 2025)). For simplification, we assumed the direct synthesis of some of the protein complexes included in the network, omitting subunit synthesis for subsequent complex conformations. This assumption was made for complexes, including LAMININ, CONDENSIN I, COHESIN, Origin of Replication and Recognition, Nuclear pore, Dynactin, Dynein, Proteasome Endopeptidase, APC/C, MCM, and RNA Polymerase II complexes.

In summary, the entire process of protein synthesis was described for 185 out of a total of 247 protein components, including individual proteins and assumed complexes. The remaining 62 proteins were divided into two groups. The first group consists of proteins considered to be present in their free form without an associated synthesis process. The second group includes proteins and complexes that undergo duplication events due to modification processes, such as phosphorylation, ubiquitination, acetylation, or dephosphorylation. A degradation event was included for each protein species in the network. The 28 end-biological processes included in the network were directly adapted from the PathwayStudio notation as “phenotype” species in CellDesigner. This species describes the influence of certain proteins within a network on external biological processes or cellular events that may not necessarily be related to genomic instability.

### 3.3. A Model for Genomic Instability Research

**Figure 2:**
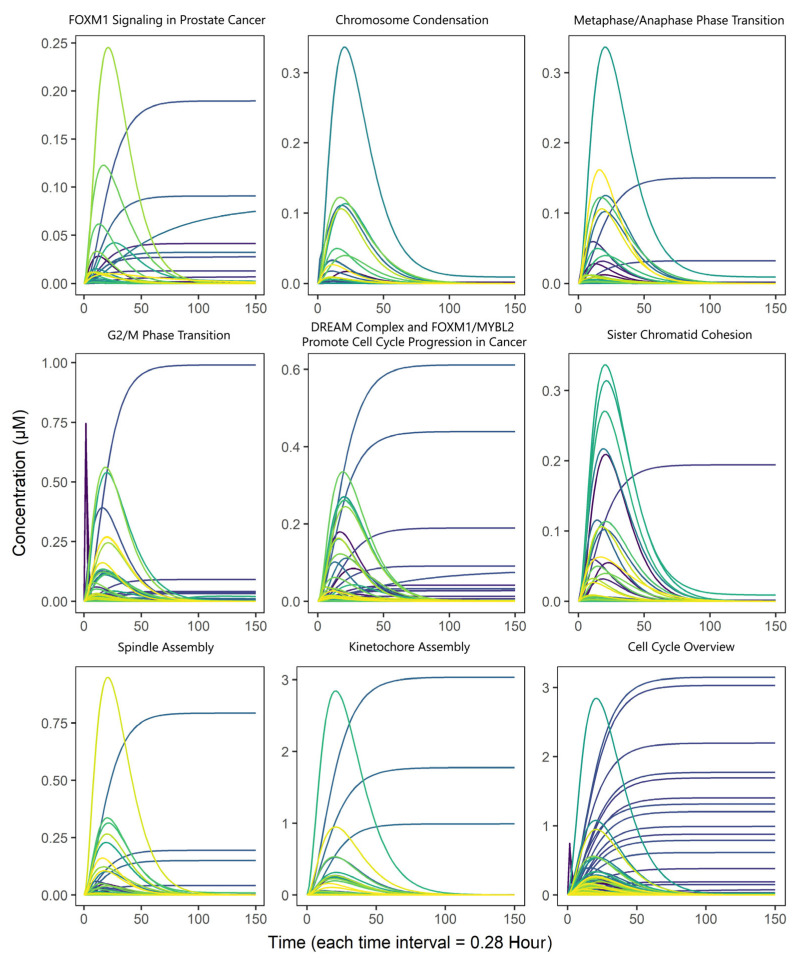
Rates of change for proteins associated with biological processes involving PLK1. Graphs represent the rates of change for a core of proteins, specifically related to the shown process, under initial conditions through 150-time intervals (each time interval representing 0.28 h). Cumulative curves without a fall in the concentration describe “phenotype” species emulating external biological processes or cell events.

We mathematically modeled the *plk1* Core Genomic Instability Network using elementary kinetics under the law of mass action, resulting in a *plk1* Core Genomic Instability Model. The model comprised a system of 716 differential equations that described the rate of change in concentration for each biological species. These equations are defined by 1030 kinetic parameters (Table S3, available at https://data.mendeley.com/datasets/xjwm5ygymn (accessed on 13 May 2025)). The simulations were performed under basal and homogeneous conditions to evaluate the dynamics of the model. This involved setting a standard initial concentration of 1.0 µM for all genes in the model (Figure 2). The CellDesigner default solver and simulation steps were implemented, and the optimal simulation time was determined by reaching a steady state.

**Figure A2:**
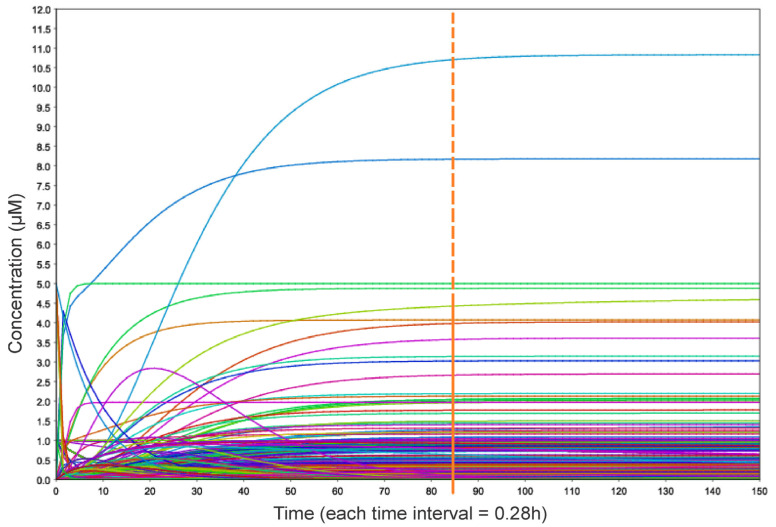
The whole simulation of the *plk1* gene regulatory network model was carried out on CellDesigner under the initial conditions. The default CellDesigner solver (SOSlib) was used to calculate the 100-steps simulation scenario. The steady state was approximately reached at the 85th interval (orange dotted line) under the initial conditions.

It is common practice to establish equivalence between simulation times and cell cycle durations when constructing mammalian-based biological models [9,17]. Among the nine biological processes considered for model construction, the Cell Cycle Overview (Table 1) had the longest duration, estimated to be 24 h, which is consistent with the typical cell cycle duration in mammals [37]. In this study, we assumed that the time at which the model reached steady state under the initial simulation conditions was equivalent to the end of the cell cycle. Based on the model simulation results under these initial conditions, we propose a hypothetical duration of 85-time intervals to represent a complete cell cycle within the model, with each time interval corresponding to a duration of 0.28 h. Consequently, we observed that the model reached a steady state at approximately 24 h [interval 85] (Appendix A, Figure A2), as expected in biological systems, which tend to maintain stability while adapting to optimal conditions for their survival.

### 3.4. Simulation Scenarios Highlight a Synergy Between PLK1 and COHESIN Regulation

Under the premise that biological species with an extensive number of interactions within the network may exert regulatory control over PLK1 involvement in genomic instability events, CDK1 (with 43 interactions), the COHESIN complex (with 23 interactions), and PLK1 (with 20 interactions) were specifically selected for the design of proper simulation scenarios and the evaluation of model dynamics. These genes were chosen based on their established connections to genomic instability events and their high connectivity among the 568 protein–protein interactions captured by the model. We established simulation scenarios that involved modulating the concentrations of genes encoded by these proteins, as outlined in the methods presented in Table 2. Furthermore, joint scenarios encompassing multiple gene variations were designed to explore potential synergistic effects.

In the case of the *cdk1* scenarios, the model did not demonstrate any significant shifts. In contrast, the *plk1* and *cohesin* evaluated scenarios (Table 2) exhibited strong similarities, indicating a synergistic effect between these two species. Additionally, scenarios with increased *plk1* concentration (Sce 11 and 12) also showed elevated COHESIN protein levels. However, whether the concentration of *cohesin* increased or decreased, it did not have any noticeable effects on PLK1 levels, highlighting a one-way synergistic effect. Therefore, COHESIN emerged as an important effector of PLK1 in the proposed *plk1* core genomic instability model. However, it is important to note that this result requires further analysis because of the regulation of the COHESIN complex, which involves three core subunits and two additional units with regulatory functions [38].

**Figure 3:**
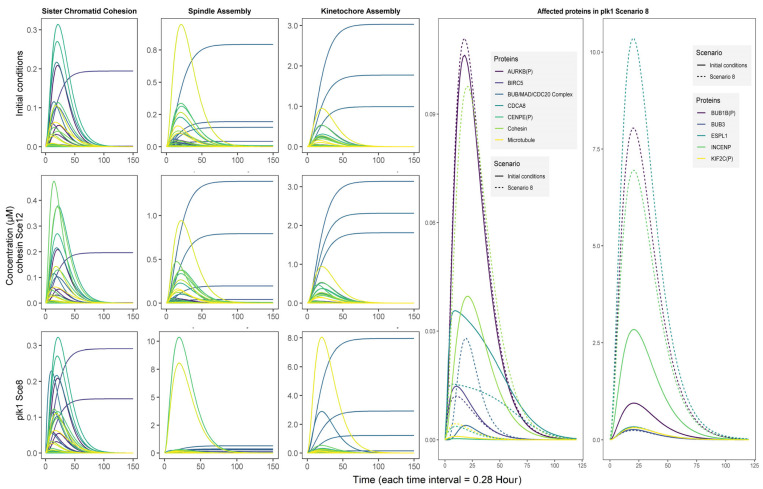
The biological processes are most affected by increased concentrations of *plk1* and *cohesin*. On the left, spindle and kinetochore assembly changes are depicted as the result of Scenario 8 simulation; similarly, shifts in protein concentration patterns were observed in multiple biological processes. In Scenario 12, sister chromatid cohesion and kinetochore assembly were modulated. The individual proteins affected by the plk1 increase are shown in the right plots.

Proteins that are affected by changes in *cohesin* concentration have diverse molecular functions, including structural elements at the microtubule-kinetochore junction, kinases, and proteins associated with important mitotic complexes, such as the chromosomal passenger complex, the mitotic checkpoint complex, and APC/C. In the case of *plk1*, the proteins that were modified due to changes in its concentration were primarily related to the mitotic checkpoint and kinetochores. As described above, similar proteins in the *cohesin* scenario were also affected by *plk1* changes. Interestingly, proteins related to carcinogenic pathways included in the model were not significantly affected by *plk1* concentration changes (Figure 3).

At the biological process level, changes in *cohesin* concentration primarily affect the kinetochore assembly and sister chromatid cohesion dynamics. Conversely, *plk1* had a more significant impact on the overall network dynamics, specifically on spindle assembly and kinetochore assembly. The observed similarities between the *plk1* and *cohesin* scenarios converge in the deregulation of the kinetochore assembly biological process (Figure 3).

### 3.5. The Role of plk1 in Genomic Instability

The gene *plk1* is usually overexpressed in tumor tissues [6]. Therefore, a scenario simulating this condition is expected to provide relevant information to better understand the involvement of *plk1* in carcinogenesis and the process of genomic instability. Taking scenario 8 as a reference (Table 1), in which *plk1* concentration increases 100-fold, we identified the biological species that changed because of an increase in *plk1* concentration, enabling their association with molecular mechanisms previously reported in the literature.

**Figure 4:**
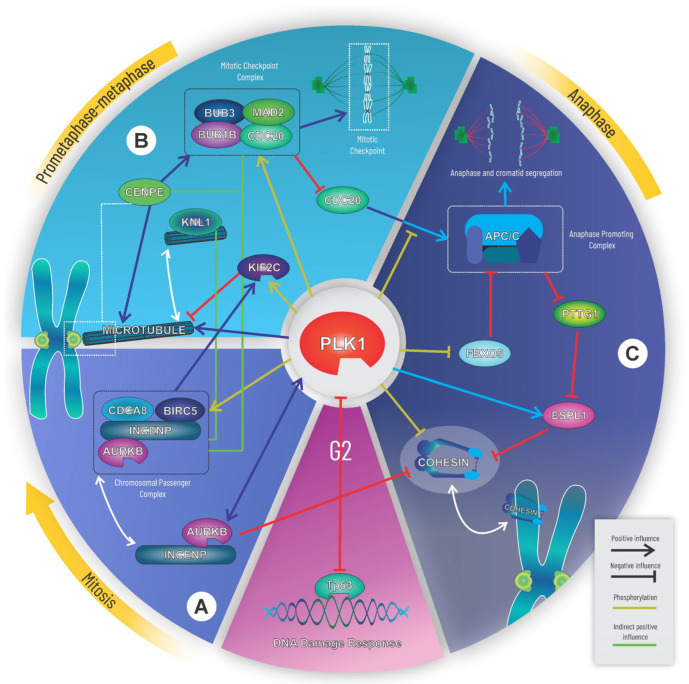
Graphical summary of the PLK1 role in genomic instability events. Simulations performed using a mass-action kinetic approach enabled the identification of three interacting circuits that can potentially induce a genomic instability event as a product of PLK1 deregulation: chromosomal passenger complex (**A**), mitotic checkpoint complex (**B**), and anaphase/cyclome promoter complex (**C**).

The simulations showed impacts on previously described interactions of *plk1*-mediated genomic instability events [6]. The affected interactions and proteins were integrated into three major circuits describing the most relevant pathways for *plk1*-mediated genomic instability events. The genes and proteins comprising these circuits are expressed and functionally expressed in a wide spectrum of tumors. The first circuit corresponds to the AURKB protein regulatory pathway and chromosomal passenger complex (Figure 4A). The second circuit describes the main events in prometaphase and metaphase, including microtubule binding to chromosomes and regulation of mitotic checkpoints (Figure 4B). The last circuit corresponds to anaphase transition and ESPL1 activity (Figure 4C). Additionally, we identified that the proteins MXD1, ZWINT, and PDS5B and the CONDENSIN I complex are affected by *plk1* increase and may serve as potential effectors of *plk1*-mediated genomic instability events. The retrieval and identification of known mechanisms strictly related to genomic instability events associated with *plk1* through computational simulations demonstrated the model’s significant potential for predicting relevant associations using contrasting scenarios (Table 1).

#### 3.5.1. First Circuit: Chromosomal Passenger Complex

According to these results, the first circuit by which PLK1 may participate in a genomic instability event in cancer is through deregulation of the chromosomal passenger complex [CPC] and the Aurora kinase B [AURKB] regulatory pathway. CPC is composed of the proteins AURKB, INCENP, Survivin [BIRC5], and Borealin [CDCA8], which are affected by the *plk1* concentration increase (Figure 3). Under normal conditions, CPC functions as a surveillance system that ensures chromosome stability and microtubule attachment. It also participates in mitosis closure processes such as chromosome decondensation and nuclear envelope reformation [39]. Individually, AURKB and BIRC5 have been linked to chromosomal instability events [36,40]. In the case of AURKB, its association with genomic instability is determined by its kinase activity during different mitotic phases, such as the mitotic spindle checkpoint [41].

#### 3.5.2. Second Circuit: Mitotic Checkpoint Complex

The second circuit contains the mitotic checkpoint complex [MCC] as its main component, which is the main effector of the mitotic spindle assembly checkpoint during mitosis. It is formed by BUB1B, BUB3, CDC20, and MAD2, which are present in the proposed model and constitute the BUB/MAD/CDC20 complex, as shown in (Figure 3). Its principal function is to inhibit the APC/C complex through direct interaction or by sequestering CDC20 to maintain checkpoint activity until the binding of microtubules to kinetochores is stable [42]. Other MCC-based interactions and regulatory pathways exist, but only the canonical interaction with APC/C is considered in the model because of its connectivity to the third circuit and its relevance in generating genomic instability [43]. Complementing the checkpoint mechanism, CENPE and KIF2C proteins were both included as signaling elements of chromosome-microtubule cohesion, which were also affected by the *plk1* concentration increment in scenario 8 (Figure 3). In addition, the proteins KNL1 and ZWINT were also affected in Sce8 and are potential markers of *plk1*-mediated genomic instability in mitotic checkpoint-centered events.

#### 3.5.3. Third Circuit: Anaphase/Cyclosome Promoter Complex

Finally, the third circuit is centered on the cohesin complex and regulation of ESPL1. Cohesin maintains sister chromatid attachment at the kinetochores and along the arms of the chromosomes, whereas ESPL1 uncouples cohesin from the chromosomes, allowing for independent sister chromatid segregation. Similarly to PLK1, ESPL1 is a licensing factor for centriole duplication [44]. Both Cohesin and ESPL1 proteins were affected by increased *plk1* concentration (Figure 3). Evidence indicates that PLK1 enhances ESPL1 activity by increasing its substrate affinity [45]. In the case of cohesin, four proteins were responsible for detaching cohesin from chromosomes. In the first removal stage, PLK1, CDK1, and AURKB detach cohesin from the chromosome arms. In the second stage, ESPL1 separates the fraction that is protected at the centromeres [38]. The second stage also allowed for the complete separation and subsequent segregation of sister chromatids, requiring the activity of other proteins and complexes in the third circuit (Figure 4C). In the model presented in this paper, we considered the participation of the APC/C complex, its inhibitor EMI1 [FBXO5], and PTTG1 [ESPL1 inhibitor], which, despite not showing relevant changes in the *plk1* overexpression scenario, are present in the model and direct the influence of PLK1 on Cohesin and ESPL1.

### 3.6. Circuit-Specific Deregulation and Prognostic Patterns in *plk1*-Driven Genomic Instability

Although our results have integrated and contributed complementary information on previously known mechanisms and even identified new potential markers for *plk1*-mediated genomic instability events, the specific regulation of the three circuits among different cancer types can lead to varying outcomes and effects. This can reinforce carcinogenic processes or induce apoptosis, as reported in the literature [46,47]. We evaluated the transcriptional profiles of 31 cancer types available in the GEPIA database, focusing on the genes comprising these circuits. The expression values of each gene in the tumor tissue were compared with those of the corresponding normal tissue (TCGA vs. GTEx). Genes were grouped by circuit for cluster analysis. For each circuit, two clusters were found to be able to divide the cancer types into those presenting the most pronounced deregulation in the circuits and those that exhibited more similar expression profiles to normal tissue.

**Figure 5:**
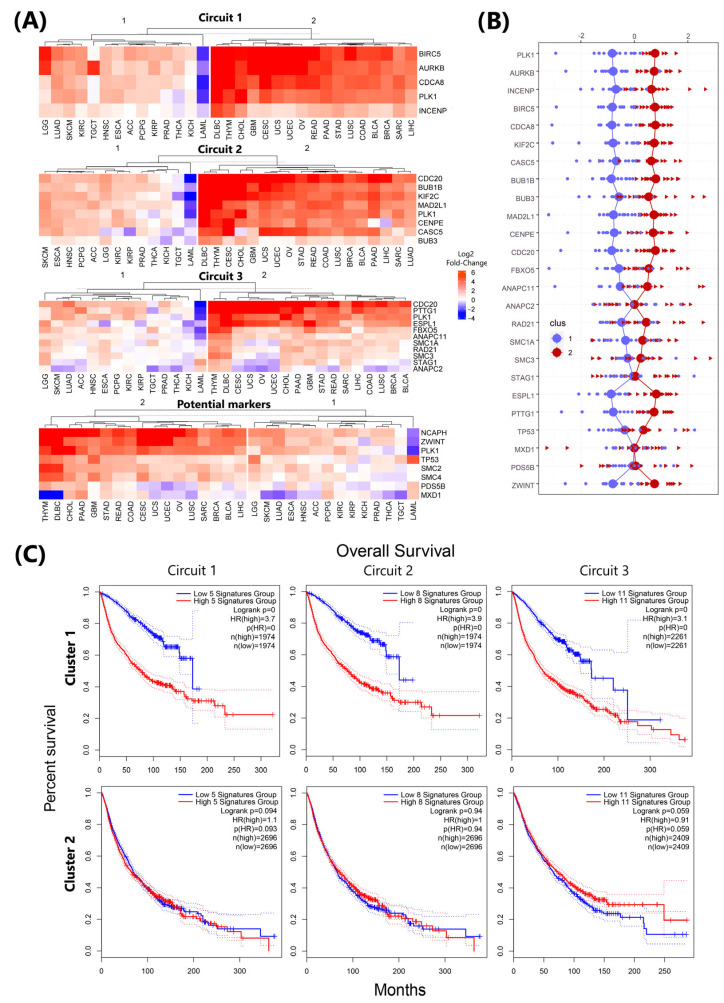
Transcriptomic profiling was conducted on the major circuits describing the most relevant pathways for *plk1*-mediated genomic instability, including known and potential new markers associated with *plk1*-mediated genomic instability. (**A**) The heatmaps display the Log2FC of TCGA vs. GTEx data, which were clustered into two groups, illustrating the optimal number to represent the data. Cluster 1 comprises cancer types with transcriptional profiles more similar to the ones exhibited by normal tissues, showing downregulation in specific genes. Cluster 2 consisted of cancer types that primarily exhibited overexpression of genes. (**B**) Distribution of data between clusters. Each point represents the Log2FC value reported for each gene by the cancer type and grouping of the clustering algorithm. (**C**) Survival analysis was performed for each circuit following the potential outcome defined by the analyzed genes in the clustered cancer types (Groups 1 and 2).

We found that out of the 31 evaluated carcinomas, the first and second circuits showed visible deregulation in 17 and 18 tumoral tissues, respectively (cluster 2 in Figure 5), with both circuits affecting mostly the same cancer types, with the sole exception of LUAD, which is clustered in the second group inside the second circuit. The dominant deregulation observed in the genes of the first and second circuits is overexpression, a phenomenon that is stronger in cancer types comprising cluster 2. The widespread and homogeneous impact on both circuits with similar expression levels in different carcinomas and the participation of multiple proteins previously associated with genomic instability led us to suggest the first and second circuits as the main mechanisms of *plk1*-mediated genomic instability events.

The third circuit exhibited similar clustering with the same cancer types observed in the first circuit (Figure 5A). However, there are certain proteins within the third circuit, such as the APC/C subunit (ANAPC2) and cohesin subunits (SMC1A, SMC3, and STAG1), that do not follow a specific pattern. While all genes comprising the first and second circuits were highly affected to a similar grade (Figure 5B), this behavior was observed only in a few genes of the third circuit, such as espl1, pttg1, and cdc20, which act as a bridge to the second circuit. Proteins that do not follow this pattern may act as important effectors in the divergence of effects observed in *plk1*-mediated genomic instability events.

In the case of new potential markers, the ZWINT and CONDENSIN I subunits NCAPH, SMC2, and SMC4 all showed similar expression levels displayed by markers in the first circuit, even sharing the same clustering of cancer types. The remaining genes PDS5B, MXD1, and the well-known gene tp53 did not follow any specific, clearly observable pattern (Figure 5A,B).

In addition, we determined the survival rates associated with each circuit by cluster. Using GEPIA database, we compared the genes comprising these circuits against the two cancer clusters identified by transcriptomic profiling (Figure 5C). Overall, for the first and second circuits, cluster 1 reported higher survival. In cluster 1, the behavior of the high and low expression groups was more differentiated in all circuits, indicating a higher survival rate for low expression profiles. In cluster 2, a similar pattern was observed for the high- and low-expression groups, with both groups sharing similar detrimental survival rates. The third circuit, for Cluster 2, showed a small divergence between the groups during the last months.

### 3.7. Real Time PCR Analysis of Key Genes Predicted to Be Associated with Genome Instability Events

**Figure 6:**
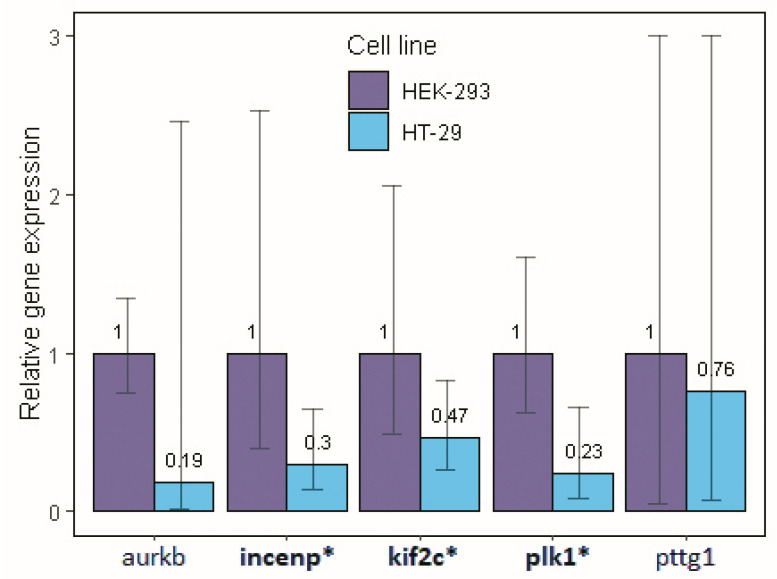
Relative expression levels of key predicted genes associated with genome instability events in HEK-293 and HT-29 cell lines. The value reported above the bars represents the calculated media for each gene; HEK-293 samples were used as a calibrator and fixed in 1.0 for relative expression calculation. The genes with statistically significant differences are marked with [*] next to the gene name.

The comparison between HEK-293 and HT-29 cell lines by qPCR showed that *plk1* expression was significantly lower in HT-29 cells (Figure 6). Thus, considering the effect that a reduction in *plk1* could have on the proposed circuits, we compared the expression levels of aurkb, incenp, kif2c, and pttg1. The suggested circuits predict that a drop in *plk1* expression levels must be followed by an increase in pttg1 and a decrease in aurkb, incenp, and kif2c expression levels. As expected, we found significant differences in the expression levels of incenp and kif2c, where the expression of both genes was lower in the HT-29 cell line. Similarly, aurkb showed a difference in the average expression between cell lines, with lower expression levels in HT-29 cells; however, this difference was not significant due to the large variation between samples. In contrast, pttg1 showed the same expression levels in both the cell lines.

The experimental validation mimicked the expected variation described by the mathematical model, showing that the expression patterns of two key genes, incenp and kif2c, were affected as a consequence of *plk1* downregulation. Both genes are important for microtubule-chromosomal dynamics and proper chromosome segregation, confirming the presence of chromosomal instability processes in the HT-29 cell line. These results are supported by genomic analyses of the studied cell line, where HT-29 cells presented a certain grade of chromosomal instability.

## 4. Discussion

Genomic instability is a hallmark of cancer, but the molecular mechanisms underlying its activation and evolution remain elusive. In this study, we constructed a *plk1* core genomic instability network in cancer by integrating previously known mechanisms and the nine essential biological processes necessary for maintaining genomic stability. The genes and their corresponding coding proteins, comprising the *plk1* core genomic instability network, were identified simultaneously in multiple cancer expression profiles and proteomic assays, highlighting that their expression and protein activities are relevant in the context of carcinogenesis for the systemic understanding of this cancer hallmark (Figure 1). Additionally, comparative analysis of the integrated *plk1*-mediated genomic instability circuits with transcriptomic data from different tumor types showed widespread impacts on the expression patterns of the three identified circuits. Furthermore, clustering analysis of the expression profiles by cancer type revealed a group of highly affected carcinomas with low survival rates, demonstrating the direct impact of the revealed molecular machinery on cancer outcomes.

### 4.1. Model Limitations and Assumptions

The developed model presents both general modeling limitations and specific constraints related to the chosen approach. Among the general limitations, structural simplification and the formulation of assumptions are necessary to define the scope of applicability. For instance, the model does not account for the influence of proteins or biological entities outside the defined network, does not capture cellular heterogeneity, and lacks an explicit representation of the temporal sequence of cellular events. Although these simplifications may limit the representation of complex biological phenomena, they enable the exploration of system behavior in a controlled and interpretable environment.

Another limitation stems from the deterministic nature of the model, which excludes the intrinsic randomness of several biological processes. This choice omits stochastic influences and may reduce the model’s capacity to reflect natural variability. Nonetheless, deterministic models are suitable for exploratory studies, particularly when data availability is limited, as is the case here.

Regarding specific limitations, a key aspect concerns the construction of a regulatory network based on large-scale data mining and natural language processing. Despite a subsequent curation process, this methodology results in varying levels of detail: genes and proteins with abundant literature were modeled with greater precision, whereas those less studied were represented more generically. To maintain model consistency, entities lacking clearly defined positive or negative influences on other components were excluded from the final network.

Moreover, the modeling of interactions was constrained by the capabilities of the software used (CellDesigner), its graphical notation, and the internal consensus within the research team. Limited information on certain mechanisms, functions, or reaction rates necessitates simplification at the level of general influence between entities. These aspects represent areas for future improvement as new experimental data become available. Similarly, some parameters were inferred by analogy to better-characterized entities or interactions, and were expected to be refined with further empirical evidence.

Finally, the main conclusions of this work focus on the best-characterized biological species, whose behavior was accurately reproduced by the model, as shown in the results section. Despite the underlying assumptions and simplifications, the model successfully captured biologically relevant behaviors and proposed potential interactions with underexplored proteins and genes. We consider this preliminary predictive capacity one of the key strengths of the *plk1* model, offering a valuable foundation for future extensions and experimental validations.

### 4.2. Deregulation Processes Leading to Genomic Instability

These three identified circuits have the potential to trigger individual genomic instability events. Indeed, proteins such as BUB1B, BUB3, CENPE, CDC20, CDCA8, ESPL1, PTTG1, and AURKB are included in these circuits and have been previously associated with genomic instability. According to the simulations, these proteins have the potential to trigger independent effects as a consequence of PLK1 deregulation. However, we also identified ZWINT, MXD1, and PDS5B as novel proteins involved in *plk1*-mediated genomic instability processes.

In the first circuit, PLK1 can impact genomic stability by affecting AURKB/CPC activity in two ways. The first is through the phosphorylation of the CPC complex protein BIRC5, which stimulates CPC activity as a complex. The second way is by activating FOXM1, which transactivates AURKB [48]. AURKB forms a protein complex with INCENP, which phosphorylates PLK1 during mitosis to activate and recruit it to the centromeres. In centromeres, PLK1 strengthens microtubule attachment to kinetochores, whereas AURKB breaks these attachments if they are unstable through KIF2C activity enhancement. Therefore, an imbalance in PLK1 or CPC/AURKB activity can lead to improper chromatid cohesion [40,49]. Additionally, AURKB and PLK1 can directly interact with the P53 factor and inhibit its activity [46,50,51].

The second circuit has the Mitotic Checkpoint Complex as its core and includes a small fraction of the checkpoint signaling pathway. The genes encoding BUB1B, BUB3, CDC20, and CENPE have been included in lists of genes associated with genomic instability or chromosomal aberrations, with the first two proteins playing essential roles in the conformation of the MCC complex, and BUB1B, particularly for its kinase activity. CENPE is a necessary protein for maintaining chromosomal stability because it directs microtubule capture and its efficient assembly in the kinetochores and participates in chromosome alignment [52]. CDC20 is the activating subunit of the APC/C complex, which promotes anaphase and chromatid segregation. KIF2C has been associated with genomic instability and some authors have proposed it as an essential protein for maintaining chromosomal stability due to its functions as a kinesin and microtubule depolymerizer [53]. PLK1 can phosphorylate BUB1B, the subunit of the MCC, and it has been shown that such modification favors the stability of the interaction between kinetochores and microtubules [54]. It can also phosphorylate KIF2C, a modification that increases its activity, and plays a role as a marker for degradation [55]. In addition, PLK1 also promotes inhibition of the APC/C complex by phosphorylation of CDC20 subunit [56].

The third circuit can lead to genomic instability owing to the coordinated cleavage of cohesin, which, in case of failure, can generate premature segregation of sister chromatids without proper assembly with microtubules. The regulation of PTTG1 is essential for this process since APC/C normally degrades PTTG1 only when the mitotic checkpoint is turned off and PLK1 induces the degradation of the APC/C inhibitor Emi1 [FBXO5] by phosphorylation [57,58]. Therefore, an increase in *plk1* expression levels may affect the regulation of the APC/C complex and promote premature degradation of PTTG1 and chromatid cleavage. In addition, overexpression of espl1 has been shown to give rise to aneuploidy in an animal model along with a tumor phenotype with high levels of genomic instability [59]. espl1 has also been reported as a genomic instability gene, as the genes coding for the RAD21 and SMC1A are subunits of the cohesin complex.

Transcription analysis carried out using the GEPIA database demonstrated a mostly homogeneous degree of deregulation in the first and second circuits, with gene overexpression being the main alteration in the analyzed carcinomas. In the case of the third circuit, there is a mixed pattern of overexpression and subexpression that makes the third circuit a relevant mechanism for understanding the different outcomes of *plk1*-mediated genomic instability. We hypothesized that the first and second circuits, centered on mitotic checkpoint regulation, microtubules, and kinetochore dynamics, are the main pathways by which *plk1* contributes to the genomic instability events observed in cancer. In both circuits, *plk1* directly interacts with different proteins related to genomic instability and acts as a master regulator.

### 4.3. plk1: Oncogene and Tumor Suppressor

Polo-like kinase 1 [*plk1*] is typically considered a proto-oncogene relevant for cell cycle progression, which is reflected in the expression profiles of different types of cancer, where it is commonly overexpressed and shows strong correlation with carcinogenesis events and checkpoint dysregulation [60,61]. The oncological potential of *plk1* has been recognized especially in gastric, breast and liver cancers and has led to efforts to develop cancer treatments and drugs based on the inhibition of its action as an oncogene [6]. However, owing in part to the large number of pathways in which PLK1 participates and the proteins it regulates, its role as a tumor suppressor in some types of cancer has recently been suggested, with experimental confirmation of this putative role [62,63,64].

The behavior of *plk1* as an oncogene or tumor suppressor is defined by the configuration provided by the tumor type and triggered dysregulations. Depending on the tumor context, *plk1* either synergizes with carcinogenic events or promotes cell death. Interestingly, the ability of *plk1* to couple with cell signaling pathways commonly associated with cancer development appears to be gathered in the non-mitotic cell cycle span, while all *plk1* functions in mitosis are generally connected to cancer through possible genomic instability events [47]. Any form of dysregulation in *plk1* expression, whether decreased or increased, has been reported to generate aneuploidies and phenotypes characteristic of genomic instability. Complete deletion of *plk1* renders non-viable cells due to drastic damage caused by improper segregation of genetic material or mitotic “slippages” [64].

The survival plots for each circuit provide evidence of how changes in not only the expression pattern of *plk1* but also the mechanism of genomic instability itself are related to poor survival rates. In the case of cluster 2, the survival rates were the same for both the low- and high-expression groups, which is consistent with the strict control required for *plk1* expression. Thus, genomic instability has emerged as an important mechanism for explaining the tumor suppressor/oncogenic role of *plk1* in cancer.

The functional separation between carcinogenic processes occurring in the non-mitotic cell cycle and genomic instability events occurring in mitosis presents an interesting dynamic that could explain why proteins that were affected by *plk1* deregulation in the simulation scenarios have functions that mainly occur during mitosis, such as CPC and MCC complex formation and the entire microtubule-kinetochore machinery assembly. This also explains why the non-mitotic processes considered for the construction of the model, such as the two FOXM1-based carcinogenesis pathways, G2/M transition, and a fraction of the Cell Cycle Overview, are not similarly affected. For instance, in the case of cdk1, it is important to highlight that for its biological activity, CDK1 requires the formation of the MPF complex and binding to Cyclin B [CCNB], which is responsible for activating and directing CDK1 activity during the G2/M transition and mitosis [65]. According to this model, the MPF dimer is one of the most regulated complexes, with multiple proteins controlling its activity. Therefore, an increase in the concentration of MPF components without increasing the proteins responsible for its activation would not result in significant shifts in the model dynamics, and MPF would likely remain inactive.

In FOXM1-based pathways with carcinogenic potential, the role played by PLK1 is less important than in mitotic pathways because it functions as an effectors of the FOXM1 transcription factor signaling cascade [32].

### 4.4. Model-Derived Hypotheses and Opportunities for Experimental Validation

Some authors have proposed that controlling chromosomal instability in a tumor could be a viable treatment option for cancer. However, genomic instability remains unpredictable. According to Bakhoum and colleagues, high levels of chromosomal instability can trigger opposite outcomes [4]. From this perspective, it is possible that the genetic heterogeneity of tumors harboring dissimilar genomic information promotes tumor growth. However, it is also probable that the damage caused by abnormal segregation of genetic information may lead to non-viable cells. Therefore, it is important to define the mechanisms that trigger one scenario or the other. Conversely, lower levels of genomic instability are usually linked to worse disease prognosis, probably because genomic changes are less drastic, enabling more stable tumor growth and a slow accumulation of genomic changes, as evidenced by tumor heterogeneity. The diversity of consequences and the different forms of genomic instability are indicators of an array of mechanisms that could also provide clues to explain the behavior of genes such as *plk1*, either as an oncogene or, conversely, a tumor suppressor

Transcriptomic profiles provide evidence that changes in *plk1* expression levels are accompanied by similar changes in the genes comprising the first and second circuits and, to some extent, the third circuit, across at least 16 different cancer types. Based on the observed behavior in the transcriptomic profiles and the presence of multiple proteins previously associated with genomic instability, we hypothesized that the first and second circuits serve as primary pathways for *plk1*-mediated genomic instability events. The third circuit is undoubtedly relevant for maintaining genomic stability; however, its transcriptomic profile demonstrates a distinct pattern from those observed in the first and second circuits.

The simulation scenario suggested that incenp and kif2c are novel markers associated with genome instability events. Additionally, supporting transcriptional analyses, the experimental validation via qPCR in HT-29 cells, was able to reproduce the expected variation described by the mathematical model, suggesting incenp and kif2c genes as novel markers associated with genome instability events. Various experiments have evaluated the effect of *plk1* knockdown on bladder cancer cells, reporting a reduction in bub1b and an increase in fbxo5 levels as a consequence of *plk1* down-regulation. These results indicate a possible alteration in circuits 2 and 3 due to *plk1* knockdown in bladder cancer [66].

We have successfully condensed a broad body of theoretical and experimental knowledge regarding *plk1* regulation in the context of genomic instability. This information was translated into a model that replicates biologically observed behaviors and serves as a platform to construct in silico scenarios using the current understanding to predict unexplored outcomes and guide or refine experimental processes. Even under baseline conditions, aimed at approximating the cellular environment during carcinogenic transformation, the simulations revealed a subset of genes and proteins within *plk1*’s regulatory neighborhood that exhibit changes in expression in response to increased *plk1* levels. Interestingly, several of these genes have not been previously associated with *plk1*-mediated genomic instability processes.

These genes include members of the cytoplasmic dynein complex (*dync1h1*, *dync1i1*, *dync1i2*, *dync1li1*, *dync1li2*, *dynlt1*, *dynlt3*, *dynll1*, and *dynll2*) and the dynactin complex (*dctn1–dctn6*), both of which are essential for the transport of cellular components, positioning of the mitotic spindle, and chromosome movement during mitosis. Additionally, structural microtubule components such as *tuba1a*, *tubb*, and *tubg1*, key constituents of the spindle apparatus, along with kinesin *kif2c* and *cohesin* regulator *pds5b*, also showed alterations under *plk1* overexpression. Although these elements are not yet directly linked to *plk1*-driven mechanisms of genomic instability, their central roles in chromosome segregation and mitotic fidelity suggest that they may represent underexplored mediators of *plk1*’s broader impact on genomic maintenance.

Taken together, these findings illustrate the capacity of the model to reveal regulatory relationships embedded within the existing literature, which remain obscure when assessed through isolated observations. Although the observed alterations in these genes emerge as a response to *plk1* overexpression within the simulated environment, it is important to emphasize that these are computational predictions. Therefore, they must be interpreted as hypotheses that require further experimental validation. Rather than confirming novel biological mechanisms, the model serves as an integrative tool that assembles and synthesizes fragmented regulatory knowledge into a coherent framework, allowing for the emergence of non-obvious interactions. This underscores the value of systems biology approaches in identifying candidate genes and interactions that could play unexplored roles in *plk1*-associated genomic instability. Importantly, the genes and molecular complexes identified here, despite their lack of direct association with *plk1*-mediated instability in the current literature, emerge from our simulations as potential focal points for future experimental studies. Targeting these candidates in validation assays may help confirm or refute their involvement, ultimately refining our understanding of the regulatory landscape shaped by *plk1* dysregulation and offering new avenues for therapeutic exploration.

To enhance the accessibility and impact of the model further, we are currently developing a web-based platform that hosts an interactive version of the *plk1* regulatory network. This platform is designed to allow researchers to modify the initial concentrations of network components, simulate regulatory perturbations, such as overexpression or knockouts, and visualize the resulting changes in system behavior in real time. Unlike traditional pathway browsers or gene enrichment tools, this interface provides a dynamic environment in which hypothesis-driven exploration of *plk1* regulatory scenarios can be conducted without requiring advanced modeling expertise.

This interactive implementation represents a significant advancement over existing tools, offering researchers a means to test conditions that are experimentally challenging to replicate, simulate the pathway-level consequences of gene-level changes, and prioritize molecular targets based on emergent system-level behaviors. Unlike static visualizations or correlation-based analyses, the mechanistic design of the model allows researchers to investigate how specific changes in *plk1* or its regulators directly impact system behavior.

In this context, it is essential to position our model relative to other platforms commonly used in molecular network analyses. Although tools such as Reactome (2025.01 release), Cytoscape (**v3.10.1)**, Pathway Commons(v12, 2023), IPA (Q1 2025 release), and GSEA (v4.3.3) offer valuable capabilities for data integration, pathway annotation, and functional enrichment, they do not provide the ability to simulate system behavior in response to quantitative perturbations. Table 3 summarizes the comparative strengths and limitations of these platforms with respect to key features, such as data consolidation, simulation capacity, scenario building, and regulatory specificity, highlighting the unique contribution of the present *plk1* model to the landscape of systems biology tools.

To elucidate from a holistic view the core circuitry associated with genome instability, in the present study, we generated a molecular network structured with the genes and proteins previously reported as important elements involved in this cancer hallmark, establishing a systemic perspective that was previously absent. Based on the three PLK1 protein-interaction circuits reported in this study (Figure 4), the model explains how dysregulation, either with increase or decreases in *plk1* levels, can generate genomic instability events in different carcinomas, emerging as a comprehensive tool for cancer research.

## 5. Conclusions

This study effectively reconstructed the core genomic instability network centered on the polo-like kinase 1 (*plk1*) gene, elucidating its critical involvement in oncogenesis. By integrating nine essential biological processes and over 1000 interactions, we established a comprehensive model to delineate the mechanisms by which *plk1* contributes to genomic instability. The obtained results indicate that *plk1* dysregulation has profound effects on chromosomal stability and correlates with reduced patient survival across multiple cancer types. The identified circuits highlight the context-dependent nature of *plk1*, wherein it typically promotes genomic instability in a variety of malignancies, yet may exhibit tumor-suppressive properties contingent upon the tumor microenvironment and specific deregulatory events. Moreover, survival analyses demonstrated a strong association between alterations in these circuits and adverse clinical outcomes, underscoring their potential as therapeutic targets.

This work represents the first installment of a broader initiative aimed at systematically reconstructing and modeling gene regulatory networks of high biological relevance. The *plk1* case illustrates the feasibility and value of this approach and serves as a template for expanding the methodology to other genes and pathways involved in key cellular processes such as cell cycle control, DNA repair, immune signaling, and metabolic regulation. Future models should be developed with similar standards of curation, modular design, and online accessibility to foster widespread use in both experimental and computational research contexts; likewise, the systemic perspective here described not only enhances the understanding of the molecular underpinnings of genomic instability but also provides a robust framework for identifying novel biomarkers and therapeutic avenues that may improve cancer management.

## Figures and Tables

**Table 1:** Simulation scenarios for the evaluation of model dynamics. Such dynamics include the model’s response to perturbations and the effects of changes in the concentration of important proteins on the molecular mechanisms and biological processes included in the model. The initial concentration (IC) in µM and 16 simulation scenarios (Sce) were considered.

	Initial Concentration Value (µM) Per Simulation Scenario (Sce)				
Gene	IC	Sce1	Sce2	Sce3	Sce4
*cdk1*	1.0	0.01	0.1	10	100
Gene	IC	Sce5	Sce6	Sce7	Sce8
*plk1*	1.0	0.01	0.1	10	100
Gene	IC	Sce9	Sce10	Sce11	Sce12
*cohesin*	1.0	0.01	0.1	10	100
Gene	IC	Sce13	Sce14	Sce15	Sce16
*cdk1, plk1, cohesin*	1.0	0.01	0.1	10	100

**Table 2:** List of biological processes in which PLK1 participates. These processes were identified using the PathwayStudio software (v12.3). Each biological process is related to a specific number of interactions implemented for model construction.

Category	Biological Process	Biological Species	Biological Interactions
Diseases	FOXM1 Signaling in Prostate Cancer	13	17
Cell processes	G2/M Phase Transition	47	65
Cell processes	Metaphase/Anaphase Phase Transition	15	22
Cell processes	Chromosome Condensation	22	36
Pathological processes	DREAM Complex and FOXM1/MYBL2 Promote Cell Cycle Progression in Cancer	36	56
Cell processes	Sister Chromatid Cohesion	27	41
Cell processes	Spindle Assembly	29	45
Cell processes	Kinetochore Assembly	49	73
Cell processes	Cell Cycle Overview	140	213
Total	9 Biological processes	378	568

**Table 3:** Capabilities of *plk1*-focused modeling versus existing pathways and functional analysis tools.

Tool/Platform	Data Consolidation (Curated or Integrative Sources)	Network Representation	Simulation Environment	Scenario Building (e.g., PLK1 Overexpression, Knockout)	Relevance to PLK1 Regulation/Genomic Instability	Limitations
PLK1 Model (this work)	High—Manual curation of PLK1-specific regulation from primary literature and reviews	Mechanistic, reaction-based (SBGN)	Yes—ODE simulation in CellDesigner	Yes—Edits to concentrations, rates, logic	Focused on PLK1 in the context of genomic instability	Requires parameter estimation; limited to encoded components
Reactome Pathway Browser	High—Expert-curated, literature-based	Mechanistic, biochemical steps	No—Static visualization only	Limited—Overlay gene expression or mutations	Contains mitotic roles of PLK1; curated context for mitosis	No dynamic behavior or causal testing
Cytoscape + ReactomeFIViz	Medium—Uses imported pathways or user-generated data	Topological; interaction & influence networks	No native simulation	Partial—Node/edge manipulation, expression mapping	Visualizes PLK1’s neighborhood; combines pathway sources	Not mechanistic; lacks temporal modeling
Pathway Commons	Very high—Aggregates >20 DBs (Reactome, Panther, HPRD)	Interaction-based (BioPAX format)	No—Data portal only	No—Requires export to SBML or Cytoscape	Useful for mining extended PLK1 network	Static; not simulation-ready without processing
IPA (Ingenuity Pathway Analysis)	High—Curated and predicted interactions from multi-omics data	Causal and regulatory network inference	No—Statistical inference only	Limited—Predicts activation/inhibition based on input	Predicts PLK1 up/downstream effects from expression data	Proprietary; no kinetic or mechanistic modeling
GSEA (Gene Set Enrichment Analysis)	None—Depends on user-defined gene sets	Not network-based; gene list comparison	No—Statistical enrichment tool	No—Identifies gene set activity in datasets	Can evaluate whether PLK1 targets or affected genes are enriched	No network structure or dynamic context

## Data Availability

The data supporting the reported results of this study can be found at the following publicly accessible link: https://data.mendeley.com/datasets/xjwm5ygymn (accessed on 13 May 2025). This repository contains relevant information regarding the model analyzed in this research.

## Supplementary Materials

The following supporting information can be downloaded at https://data.mendeley.com/datasets/xjwm5ygymn (accessed on 13 May 2025).
